# Recreational soccer and basketball improve anthropometric, body composition and health-related outcomes in overweight and obese young adults: A randomized multi-arm study

**DOI:** 10.5114/biolsport.2025.139859

**Published:** 2024-08-30

**Authors:** Qi Xu, Rui Miguel Silva, Piotr Zmijewski, TingYu Li, Dong Ma, LiuXi Yang, GuiYang Liu, Filipe Manuel Clemente

**Affiliations:** 1 Gdansk University of Physical Education and Sport, 80-336 Gdańsk, Poland; 2 Sport Physical Activity and Health Research & Innovation Center (SPRINT), Viana do Castelo, Portugal; 3 Escola Superior Desporto e Lazer, Instituto Politécnico de Viana do Castelo, Rua Escola Industrial e Comercial de Nun’Álvares, 4900-347 Viana do Castelo, Portugal; 4 Jozef Pilsudski University of Physical Education in Warsaw, Warsaw, Poland; 5 School of Athletic Performance, Shanghai University of Sport, 200438, Shanghai, China; 6 Physical Education and Health Education, Udon Thani Rajabhat University 64 Thaharn Road, Muang, Udon Thani 41000, Thailand

**Keywords:** Football, Physical exercise, Obesity, Body mass index, Blood pressure, Heart rate

## Abstract

This study aimed to investigate the effects of 8-week programmes based on recreational soccer (SCG) and basketball (BCG) conditioned games compared to self-exercise (SECG) and inactive (ICG) control groups on anthropometry, body composition, resting heart rate (RHR), and blood pressure among sedentary overweight and obese men and women. The study included 90 volunteers (aged 19.8 ± 1.5 years; 45 women) who were assessed twice, before and after the intervention, for the waist circumference (WC), body mass index (BMI) and body composition (skinfold sum and %body fat), RHR and systolic (BPs) and diastolic (BPd) blood pressure. Members of the experimental groups participated in three training sessions per week, while the control groups either maintained their routines or engaged in self-regulated exercises. Time × group interactions were found for BMI (p < 0.001; = 0.339), body fat (p < 0.001; = 0.317), WC (p < 0.001; = 0.429), skinfold sum (p < 0.001; = 0.818), RHR (p < 0.001; = 0.572), BPs (p < 0.001 = 0.534) and BPd (p < 0.001; = 0.633). Between-group analysis revealed greater improvements in BMI (p < 0.05), body fat (p < 0.05), WC (p < 0.05), skinfold sum (p < 0.05), RHR (p < 0.001), and BPd (p < 0.001) in the SCG and BCG compared to the SECG and ICG. No differences in improvements between the groups were observed. No time × group interactions were observed for sex (p > 0.05). The results suggest that intervention programmes based on recreational games such as basketball or soccer are effective in improving anthropometric characteristics, body composition, and cardiovascular health in sedentary overweight and obese men and women, and are independent of sex. The effectiveness is greater when accompanied by a more significant reduction in energy intake. Engaging in self-controlled physical activity proved to be beneficial when compared to remaining inactive, despite changes in dietary intake.

## INTRODUCTION

Overweight and obesity has been emerging as a global health concern, with greater negative implications for sedentary individuals [1]. The prevalence of overweight and obesity is a consequence of a sedentary lifestyle, poor nutrition and hydration habits, and other contextual and environmental factors [2]. As a result, sedentary routines and obesity, and their associated health problems, have received attention in the field of public health and clinical research [3]. One of the major ways to minimize their negative health impact on sedentary and obese individuals is physical activity.

Excess weight, particularly in the form of visceral fat, is strongly associated with the risk of hypertension development [4, 5]. The link between sedentarism, overweight, and obesity, and the development of hypertension is well established, as excess body weight places additional strain on the cardiovascular system, leading to higher blood pressure levels [6]. Moreover, the inflammatory and metabolic changes associated with obesity contribute to the susceptibility to heart-related diseases, such as coronary artery disease and myocardial infarction [7]. This results in the heart having to work harder to pump blood throughout the body, leading to elevated blood pressure levels. Over time, persistent high blood pressure can damage blood vessels and contribute to fat accumulation in the arteries, narrowing them and increasing the risk of heart-related diseases [8].

Physical activity plays a pivotal role in the prevention and minimization of obesity and comorbidities, with extensive research alerting to its very positive effects on sedentary and obese individuals’ overall health [9, 10]. For instance, the regular practice of different forms of physical activity has been consistently linked to positive changes in anthropometrics, body composition, and heart-related diseases, thus enhancing life quality [9, 10]. Experimental interventions that promoted physical activity have shown the potential to mitigate the negative impacts of obesity and sedentarism [3, 11, 12]. It is often observed that structured training programmes for these populations are typically based on single exercise modalities such as jogging, cycling, or resistance training [13]. While effective, such activities may not motivate sedentary individuals, particularly those who prefer social engagement and team dynamics [14]. Therefore, recreational team sports, such as soccer and basketball, have gained popularity as more enjoyable forms of physical activity that simultaneously offer social interaction and positive health adaptations [15].

For instance, a previous systematic review and meta-analysis suggested the effectiveness of recreational soccer in reducing body fat in sedentary populations [16]. Additionally, earlier systematic reviews have indicated the beneficial effects of SSGs involving recreational soccer on untrained men and women from hypertensive populations, despite the presence of high levels of heterogeneity [17]. However, concerning resting heart rate (RHR), an original study did not find evidence supporting its benefits [18]. RHR can serve as a valuable health marker, as higher values (> 80 bpm) are significantly associated with an increased risk of end-stage renal disease, for example [19].

Despite the increasing interest in recreational team sports, the existing evidence still lacks a thorough comparison between different recreational team sports, such as soccer and basketball, for sedentary, overweight, and obese individuals. Furthermore, the majority of research on this topic focuses on older populations [20] and children [21], with limited investigation into young adults. Finally, there is a notable lack of research comparing the effects and interactive impact of sex factors on these adaptations.

Comprehending the influence of various popular recreational team sports, such as soccer and basketball, on anthropometric measurements, body composition, and essential health parameters among sedentary, overweight, and obese young adults holds significant importance. This study aimed to contribute evidence regarding the effects on this demographic. Establishing a foundation of evidencebased insights that endorse recreational team sports as a pleasurable avenue for promoting systematic physical activity can support the effectiveness of intervention in mitigating sedentary behaviour and addressing obesity. Additionally, investigating potential gender-related effects offers a distinctive perspective for sports orientation.

For the aforementioned reasons, this study aimed to compare the effects of 8-week participation in recreational soccer (SCG) and basketball (BCG) conditioned games with self-exercise (SECG) and inactive (ICG) control groups. The study purpose was to assess the effect of intervention on anthropometry, body composition, RHR, and blood pressure among sedentary overweight and obese men and women. As previous studies may suggest [22], our hypothesis was that significant improvements in waist circumference (WC), body mass index (BMI), body composition (sum of skinfolds and % body fat), RHR, and systolic (BPs) and diastolic (BPd) blood pressure would be observed within the experimental groups. Additionally, we hypothesized that significant improvements would be identified in the experimental groups compared to the control groups.

## MATERIALS AND METHODS

### Study design

**FIG. 1:**
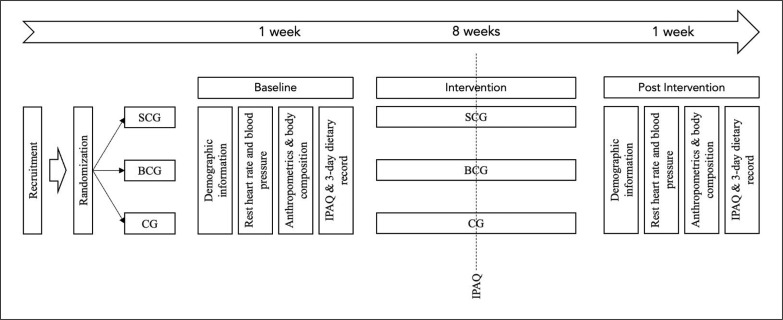
Study design. SCG: soccer conditioned games; BCG: basketball conditioned games; CG: control group (including both self-exercise and inactive control groups).

This study is a multi-arm-controlled study, composed of two experimental groups (recreational soccer conditioned games group, SCG; and recreational basketball conditioned games group, BCG) and two control groups (self-exercise control group, SECG; and inactive control group, ICG). After the participants’ recruitment, they were randomly assigned to one of the groups, before the first assessment. The randomization was made by sealed opaque envelopes by a nonrelated person with the researchers’ team. Recruitment was performed using social media announcements, focused on sedentary young adults with overweight (25.0–29.9 kg/m^2^) or obesity (> 30 kg/m^2^). Participants were assessed twice (before and after the 8-week training intervention), for their anthropometry, body composition, and health-related outcomes (Figure 1). Assessors were blind to the group assignment. The study protocol was a priori analysed and approved by the Chendu Institute of Physical Education with the code 124/2023. Moreover, participation was voluntary and the participants signed a consent form and were informed in advance about the risks and benefits and the possibility to withdraw from the study without any penalty. The study followed the ethical guidelines of the Declaration of Helsinki.

### Participants

A priori sample size calculation was conducted to identify the recommended sample for the experiment. Given an effect size of 0.13 [23], and considering four groups, a power of 0.85, and one covariate (sex), the software G∗Power (version 3.1.9.6., Heinrich-Heine-Universität Düsseldorf, Düsseldorf, Germany) determined an advisable participant size of 87 individuals.

Eligibility criteria for participants included: (i) being sedentary (with a body mass index (BMI) of 25.0–29.9 kg/m^2^) or obese (with a BMI exceeding 30 kg/m^2^); (ii) absence of chronic diseases such as hypertension or diabetes; and (iii) commitment to participating in at least 85% of the intervention programme and attending both assessment sessions. The exclusion criteria comprised: (i) missing any of the assessment sessions; (ii) failing to meet the requirement of attending a minimum of 85% of the training sessions; (iii) having a BMI below the standardized threshold set in the inclusion criteria; and (iv) experiencing chronic diseases or injuries at the time of the intervention. Following the initial recruitment, out of the 98 volunteers expressing interest in the experiment, 90 met the eligibility criteria and were subsequently assigned to the groups. Both the SCG and BCG comprised 30 participants each, consisting of 15 men and 15 women in each group.

Control groups were categorized as self-exercise (n = 10; for those who started engaging in physical exercise at least three times a week without supervision – being controlled by means of the International Physical Activity Questionnaire (IPAQ)) [24] and inactive (n = 20; for individuals maintaining their sedentary lifestyle without engaging in physical exercise). In total, the participants had an average age of 19.8 ± 1.5 years, and comprised 45 men and 45 women. The body mass was 75.1 ± 8.5 kg, and height 1.68 ± 0.07 cm. After the 8-week intervention, participants in the SCG demonstrated an adherence rate of 95%, while those in the BCG had an even higher adherence rate of 97%. There were no recorded dropouts after the allocation to groups. Table 1 provides the descriptive statistics of the main demographic characteristics for each group.

**TABLE 1:** Demographic characteristics (expressed as mean ± standard deviation) of each group.

	SCG	SCG	BCG	BCG	SECG	SECG	ICG	ICG

	Men	Women	Men	Women	Men	Women	Men	Women

N	15	15	15	15	5	5	10	10
Age (years)	19.7 ± 1.4	20.1 ± 1.5	20.1 ± 1.5	19.7 ± 1.4	20.0 ± 1.9	19.8 ± 1.8	19.9 ± 1.7	19.4 ± 1.3
Height (cm)	1.73 ± 0.04	1.64 ± 0.04	1.74 ± 0.04	1.61 ± 0.04	1.74 ± 0.01	1.63 ± 0.04	1.74 ± 0.04	1.61 ± 0.04
Body mass (kg)	80.7 ± 6.2	69.5 ± 4.7	82.2 ± 5.7	68.1 ± 4.3	78.2 ± 7.8	69.0 ± 6.7	82.4 ± 8.4	69.5 ± 5.2

SCG: soccer conditioned games; BCG: basketball conditioned games; SECG: self-exercise control group; ICG: inactive control group; N: number of participants

### Training and lifestyle intervention

After being assessed for the first time, and before starting the intervention, all the participants (those enrolled in experimental and those enrolled in control groups) received a 2-hour lecture about the relevance of a physically active lifestyle for health and functional nutrition. This lecture aimed to outline the benefits of physical exercise in reducing the risk of premature death and managing non-communicable diseases. Additionally, it provided guidelines for incorporating effective and time-efficient exercise routines. Furthermore, it offered tips for dietary management, including controlling energy intake and integrating an active lifestyle into daily activities, such as taking regular active breaks during work or preparing homemade meals to monitor intake. Nevertheless, participants in both the experimental and control groups were not provided with any dietary plans, individual physical exercise regimens, or guidance.

Those assigned to experimental groups participated in three training interventions a week, over 8 weeks. The sessions of SCG consisted of a standardized 5-minute warm-up, followed by conditioned games of 2 v 2 and 4 v 4 formats of play without a goalkeeper and using small goals (2 × 1 m). The formats of play were done in 25 × 15 and 35 × 20 m, respectively. In the sessions of the 2 v 2 format, the participants had 4 repetitions of 3 minutes, interspaced by 2-minute rests, while in the 4 v 4 format, they had four sets of 5 minutes, with 2-min rests. The games had no offside rules, and many balls were positioned around the field for faster ball repositioning.

In the case of BCG, we employed both 3 v 3 and 5 v 5 formats. These games utilized small baskets with a height of 2.60 m. The 3 v 3 variant was played within a quarter of the court, whereas the 5 v 5 format covered half of the court. Each format underwent four repetitions; however, the 3 v 3 games were played for 3 minutes per repetition, while the 5 v 5 games extended to 5 minutes. A 2-minute rest interval was incorporated between each repetition for both formats.

After the lecture, a subset of the control group spontaneously embraced an active lifestyle and participated in physical exercise. Individuals within the SECG who chose to engage in self-directed exercise did so voluntarily and independently, devoid of external influence or guidance. However, periodic assessments were conducted three times during the study, monitoring self-reported weekly physical activity using the short-version International Physical Activity Questionnaire. These responses facilitated the classification of SECG participants into active and inactive categories.

Active SECG participants enjoyed the autonomy to self-prescribe activities, such as running, swimming, or resistance training, among others. Despite this, assessments ensured ongoing monitoring of their reported weekly physical activity. Conversely, participants in the control group were mandated to partake in self-prescribed activities, with a prohibition on availing of personal training services or attending physical classes. This condition was established to uphold individual responsibility for voluntary exercise endeavours.

### Methodological procedures

During both assessments (pre- and post-intervention), participants underwent measurements on Mondays following a 48-hour rest period as requested. The anthropometric and physiological assessments commenced at 7:00 a.m. under fasting conditions, within a room maintained at a temperature of 22ºC and a relative humidity of 50%. The evaluation process was initiated with the completion of a demographic questionnaire and the short-version IPAQ [24]. Subsequently, participants were examined for RHR and blood pressure, followed by anthropometric measurements. The order of the participants was maintained to ensure consistency between the two assessment sessions. Participants were evaluated in small groups, consistently moving through the tests in the same order each time.

### Resting heart rate and blood pressure

RHR was assessed using a 1 Hz heart rate monitor (Polar RS400, Kempele, Finland) following a 15-minute period of rest in the supine position in a quiet space [25]. The RHR point was determined by calculating the average of the three minimal beats per minute recorded. The blood pressure was also measured in the supine position, two assessments being recorded (one for each arm) consecutively and using the average of them for systolic (BPs) and diastolic (BPd) blood pressure. The blood pressure was measured using a validated automated upper arm home blood-pressure monitor (BP7450, Omron Healthcare) [26]

### Anthropometry

Two experienced assessors, proficient in anthropometric measurements, began evaluating the participants’ stature and body mass. The assessors were certified in physical education and sports sciences and had completed workshops in anthropometric assessments. With over three years of experience, these assessments are a routine part of their practice. Participants were instructed to wear a T-shirt, shorts, and no socks during the measurements. Standing with their backs against the height rule and looking straight ahead to align the Frankfort plane horizontally, participants were positioned for accurate measurements. The assessor, located in front of the participant, then positioned the stadiometer marker (ADE MZ10042, ADE, Germany).

For body mass measurement assessment, participants, facing forward, stood at the centre of the platform. Measurements were conducted using an electronic flat scale (Model 813, SECA, Germany).

WC was assessed by having the participant stand upright with their feet close together, pinpointing the midpoint between the top of the hip bone and the bottom of the ribcage. Subsequently, a nonelastic measuring tape (Lufkin W606PM, Mexico) was encircled around the waist parallel to the floor, ensuring a snug but not overly tight fit. The measurement was taken at the end of a normal breath, and the average of two measurements was recorded.

Skinfold measurements were obtained for the biceps, triceps, subscapular, and suprailiac sites [27] utilizing the Harpenden skinfold caliper (British Indicators, Ltd., London, UK) with precision to the nearest 0.1 mm. The measurements were taken on the right side of the body, with the subject standing in a relaxed posture. For the biceps, the measurement was obtained at the mid-point between the acromion and the radial head. Triceps measurements were taken at the midpoint between the acromion and olecranon processes. The subscapular skinfold was measured at a diagonal fold, just below the inferior angle of the scapula. Lastly, the suprailiac measurement was taken at the iliac crest fold, along the natural angle of the iliac crest. Each measurement was repeated twice, with the average recorded for further data treatment, and the sum of these skinfold measurements was extracted for data analysis and to estimate body fat as previously validated for Chinese men and women adults [27].

### Three-day dietary record

To analyse potential variations in participants’ dietary behaviours over the period of the study, a three-day dietary record (24-hour Dietary Record) was administered both before and after the intervention. The recorded days encompassed two consecutive weekdays and one weekend day [28]. A customized form was devised to document all foods consumed by participants during this three-day period. Subsequently, these foods were categorized into 14 broad food groups and 12 types of nutrients based on the Selection and Preparation of Food for Residents in China Dietary Guidelines [28]. The daily nutrient intake of participants was determined by crossreferencing the food codes with the China Food Composition Database. In cases where a particular food was not listed in the database, a similar food item was substituted for the purpose of calculating nutritional values [28]. The daily caloric intake served as the outcome.

### Statistical procedures

Data normality and homogeneity were confirmed through the Kolmogorov-Smirnov test (*p* > 0.05) and Levene’s test (*p* > 0.05), respectively. Descriptive statistics, including mean and standard deviation, were presented. For both within- and between-group comparisons, repeated measures analysis of covariance (ANCOVA) adjusted for sex was employed. ANCOVA effect size was reported using partial eta squared, while pairwise comparisons were expressed using Cohen’s d. Post hoc analysis utilized Bonferroni tests. Furthermore, a two-way ANOVA was employed to examine the interactions between group and sex concerning the contrast analysis (post-pre) for the various outcomes analysed. The Pearson product-moment correlation test was conducted to analyse the relationships between the post-pre variations in energy intake and changes in physical fitness among both men and women. Thresholds for partial eta squared were established as follows [29]: < 0.01, small; > 0.06, moderate; and > 0.14, large. Additionally, criteria for Cohen’s d were defined as follows [30]: d > 0.2, small; d > 0.5, medium; and d ≥ 0.8, large. The magnitude of Pearson r correlations was interpreted as follows [31]: r < 0.1, trivial; r > 0.3, moderate; r > 0.5, large; r > 0.7, very large; > 0.9, nearly perfect. All statistical analyses were conducted using SPSS software (version 29.0.0., IBM SPSS Statistics, Armonk, NY: IBM Corp), with significance set at *p* < 0.05.

## RESULTS

## Baseline assessments

No differences at baseline were found between groups considering BMI (*p* = 0.359; = 0.037), body fat (*p* = 0.455; = 0.030), WC (*p* = 0.537; = 0.025), skinfold sum (*p* = 0.724; = 0.015), RHR (*p* = 0.096; = 0.071), BPs (*p* = 0.313; = 0.040) and BPd (*p* = 0.202; = 0.052).

## Pre-to-post-training variations

Table 2 shows the descriptive statistics of the two experimental and the two control groups over the periods of the experiment (pre-, and post-intervention). Significant interaction effects were found between time and group on BMI *p* < 0.001), body fat (*p* < 0.001), WC (*p* < 0.001), skinfold sum (*p* < 0.001), RHR (*p* < 0.001), BPs (*p* < 0.001) and BPd (*p* < 0.001).

**TABLE 2:** Descriptive statistics (mean ± standard deviation) and inferential statistics for the within- and between-group comparisons for the overall participants.

	SCG (n = 30)	BCG (n = 30)	SECG (n = 10)	ICG (n = 20)	Mixed repeated measures ANCOVA
**BMI (kg/m^2^)**					
Pre	27.7 ± 1.4	28.0 ± 2.0	27.1 ± 1.7	28.3 ± 1.8	time × group × sex (F = 0.819; *p* = 0.487; = 0.029)
Post	25.1 ± 1.6^#^	25.4 ± 2.1	24.7 ± 2.0	26.8 ± 1.8	time × group (F = 14.030; *p* < 0.001; = 0.339)
Post-pre (%D)	ß -9.4	ß -9.3	ß -8.9	ß -5.3	time × sex (F = 3.458; *p* = 0.067; = 0.040)

**Body fat (%)**					
Pre	32.6 ± 1.8	32.8 ± 2.6	31.7 ± 2.0	33.1 ± 2.2	time × group × sex (F = 0.172; *p* = 0.915; = 0.006)
Post	29.3 ± 1.8^#^	29.7 ± 2.5	28.8 ± 2.3	31.3 ± 2.2	time × group (F = 12.707; *p* < 0.001; = 0.317)
Post-pre (%D)	ß -10.1	ß -9.5	ß -9.1	ß -5.4	time × sex (F = 33.623; *p* = 0.060; = 0.042)

**WC (cm)**					
Pre	92.8 ± 5.3	91.7 ± 5.8	90.6 ± 6.0	93.4 ± 6.0	time × group × sex (F = 0.097; *p* = 0.961; = 0.004)
Post	87.7 ± 5.2^#^	87.4 ± 5.7	87.1 ± 5.8	92.5 ± 5.5	time × group (F = 20.512; *p* < 0.001; = 0.429)
Post-pre (%D)	ß -5.5	ß -4.7	ß -3.9	ß -1.0	time × sex (F = 0.017; *p* = 0.898; < 0.001)

**Skinfold Sum (mm)**					
Pre	76.5 ± 14.9	78.3 ± 9.4	75.3 ± 11.2	79.9 ± 13.6	time × group × sex (F = 0.098; *p* = 0.961; = 0.004)
Post	68.4 ± 15.3^#^	70.7 ± 9.3	71.0 ± 11.3	77.7 ± 13.6	time × group (F = 122.724; *p* < 0.001; = 0.818)
Post-pre (%D)	ß -10.6	ß -9.7	ß -5.7	ß -2.8	time × sex (F = 0.374; *p* = 0.542; = 0.005)

**Resting HR (bpm)**					
Pre	83.4 ± 3.7	84.8 ± 3.5	86.4 ± 2.8	84.9 ± 2.9	time × group × sex (F = 0.061; *p* = 0.980; = 0.002)
Post	79.0 ± 3.5^#^	80.1 ± 3.7	83.0 ± 3.0	84.1 ± 3.2	time × group (F = 36.475; *p* < 0.001; = 0.572)
Post-pre (%D)	ß -5.3	ß -5.5	ß -3.9	ß -0.9	time × sex (F = 0.672; *p* = 0.415; = 0.008)

**BPs (mmHg)**					
Pre	135.6 ± 6.2	132.4 ± 7.1	132.8 ± 5.5	133.4 ± 7.9	time × group × sex (F = 0.947; *p* = 0.422; = 0.033)
Post	132.1 ± 5.5^#^	128.9 ± 6.5	127.6 ± 4.6	134.2 ± 6.7	time × group (F = 31.329; *p* < 0.001; = 0.534)
Post-pre (%D)	ß -2.6	ß -2.6	ß -3.9	Ý +0.6	time × sex (F < 0.001; *p* = 0.985; < 0.001)

**BPd (mmHg)**					
Pre	85.8 ± 5.9	84.0 ± 5.5	81.5 ± 5.4	84.2 ± 5.6	time × group × sex (F = 1.744; *p* = 0.164; = 0.060)
Post	81.8 ± 5.8^#^	79.5 ± 5.0	77.1 ± 5.8	84.5 ± 5.4	time × group (F = 47.148; *p* < 0.001; = 0.633)
Post-pre (%D)	ß -4.7	ß -5.4	ß -5.4	Ý +0.4	time × sex (F = 1.059; *p* = 0.306; = 0.013)

SCG: soccer conditioned games; BCG: basketball conditioned games; SECG: self-exercise control group; ICG: inactive control group; BMI: body mass index; WC: waist circumference; HR: heart rate; BPs: systolic blood pressure; BPd: diastolic blood pressure; #: significantly (p < 0.05) different from pre assessment

SCG significantly improved the BMI (-9.4%; *p* < 0.001; d = 1.728), body fat (-10.1%; *p* < 0.001; d = 1.866), WC (-5.5%; *p* < 0.001; d = 0.975), skinfold sum (-10.6%; *p* < 0.001; d = 0.512), RHR (-5.3%; *p* < 0.001; d = 1.222), BPs (-2.6%; *p* < 0.001; d = 0.514) and BPd (-4.7%; *p* < 0.001; d = 0.683).

BCG significantly improved the BMI (-9.3%; *p* < 0.001; d = 1.233), body fat (-9.5%; *p* < 0.001; d = 1.211), WC (-4.7%; *p* < 0.001; d = 0.731), skinfold sum (-9.7%; *p* < 0.001; d = 0.804), RHR (-5.5%; *p* < 0.001; d = 1.287), BPs (-2.6%; *p* < 0.001; d = 0.486) and BPd (-5.4%; *p* < 0.001; d = 0.801).

SECG significantly improved the BMI (-8.9%; *p* < 0.001; d = 1.208), body fat (-9.1%; *p* < 0.001; d = 1.259), WC (-3.9%; *p* < 0.001; d = 0.584), skinfold sum (-5.7%; *p* < 0.001; d = 0.370), RHR (-3.9%; *p* < 0.001; d = 1.141), BPs (-3.9%; *p* < 0.001; d = 0.894) and BPd (-5.4%; *p* < 0.001; d = 0.763).

ICG significantly improved the BMI (-5.3%; *p* < 0.001; d = 0.797), body fat (-5.4%; *p* < 0.001; d = 0.783) and skinfold sum (-2.8%; *p* < 0.001; d = 0.165). However, no significant differences were observed for WC (-1.0%; *p* = 0.053; d = 0.144), RHR (-0.9%; *p* = 0.123; d = 0.261), BPs (+0.6%; *p* = 0.171; d = 0.087) and BPd (+0.4%; *p* = 0.532; d = 0.054).

Between-group analysis revealed that the improvements (post-pre) in BMI of SCG (mean improvement: -2.6%) and BCG (mean improvement: -2.5%) were significantly greater than in SECG (*p* = 0.004 and *p* = 0.011, respectively) and ICG (*p* = 0.002 and *p* = 0.008, respectively). No significant differences were observed between the SCG and BCG (*p* > 0.05) and the SECG and ICG (p > 0.05).

The post-intervention enhancements in body fat percentage for the SCG (mean improvement: -3.4%) and BCG (mean improvement: -3.1%) were significantly greater than in SECG (*p* = 0.002 and *p* = 0.021, respectively) and ICG (*p* < 0.001 and *p* = 0.015, respectively). No significant differences were observed between the SCG and BCG (*p* > 0.05) and the SECG and ICG (*p* > 0.05).

The SCG (mean improvement: -5.1%) and BCG (mean improvement: -4.2%) showed significantly greater reduction in WC compared to the SECG (p < 0.001 and *p* = 0.045, respectively) and ICG (*p* < 0.001 and *p* < 0.001, respectively). No significant differences were observed between the SCG and BCG (*p* > 0.05) and the SECG and ICG (p > 0.05).

Significant improvements (post-pre) in skinfold sum were evident in both the SCG (mean improvement: -8.1%) and BCG (mean improvement: -7.6%). These improvements were significantly greater compared to the SECG (*p* < 0.001 and *p* < 0.001, respectively) and ICG (*p* < 0.001 and *p* < 0.001, respectively). No significant differences were observed between the SCG and BCG (*p* > 0.05) and the SECG and ICG (*p* > 0.05).

The improvements (post-pre) in RHR of the SCG (mean improvement: -4.5%) and BCG (mean improvement: -4.7%) were significantly greater than in the SECG (*p* < 0.001 and *p* < 0.001, respectively) and ICG (*p* < 0.001 and *p* < 0.001, respectively). No significant differences were observed between the SCG and BCG (*p* > 0.05) and the SECG and ICG (*p* > 0.05).

The post-pre improvements in BPs for both the SCG (mean improvement: -3.5%) and BCG (mean improvement: -3.5%) were significantly greater compared to the ICG (*p* = 0.019 and *p* = 0.019, respectively). No significant differences were observed between the SCG, BCG and SECG (*p* > 0.05) and between the SECG and ICG (p > 0.05).

The improvements (post-pre) in BPd of the SCG (mean improvement: -4.0%) and BCG (mean improvement: -4.5%) were significantly greater than in the SECG (*p* = 0.010 and *p* < 0.001, respectively) and ICG (*p* < 0.001 and *p* < 0.001, respectively). No significant differences were observed between the SCG and BCG (*p* > 0.05) and the SECG and ICG (*p* > 0.05).

**FIG. 2:**
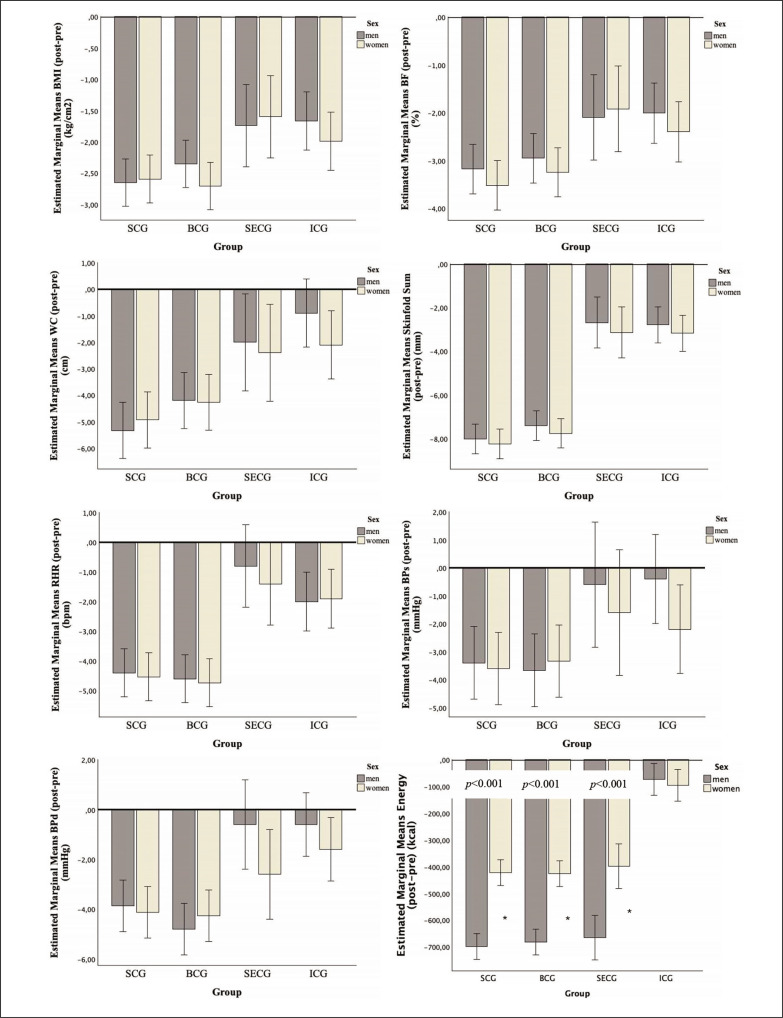
Estimated marginal means for pre-post values of anthropometric, body composition, and physiological variables, taking into account the groups and sex. Legend: WC: waist circumference; BMI: body mass index; BF: body fat; HR: heart rate; BPd: diastolic blood pressure; BPs: systolic blood pressure. SCG: soccer conditioned games; BCG: basketball conditioned games; SECG: self-exercise control group; ICG: inactive control group *: significant differences between sexes.

In Figure 2, the variations (post-pre) in anthropometric measurements, body composition, and health-related outcomes among men and women are shown for both the experimental and control groups. No significant interactions were found between group and sex (*p* > 0.05) regarding the analysed variables.

**FIG. 3:**
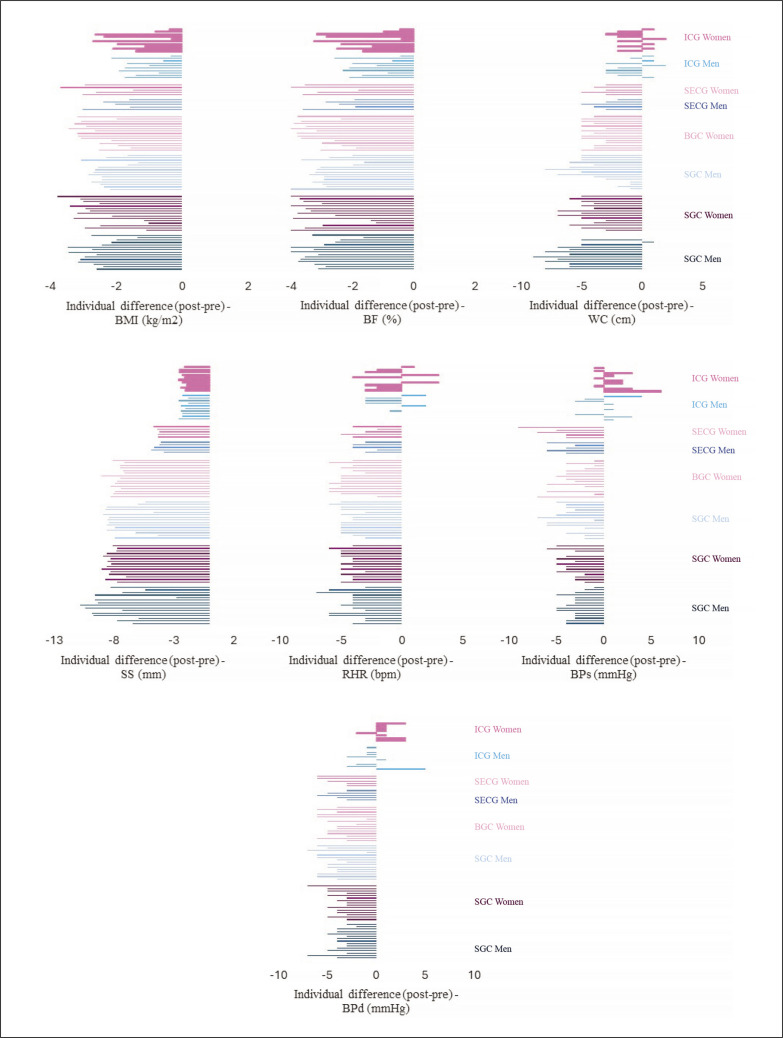
Individual variations for pre-post values of anthropometric, body composition, and physiological variables, taking into account the groups and sex. Legend: RHR: resting heart rate; BPs: systolic blood pressure; BPd: diastolic blood pressure; SS: skinfold sum; WC: waist circumference; BMI: body mass index; BF: body fat.

Figure 3 shows the intra-individual differences (post-pre) in anthropometric, body composition, and physiological outcomes analysed. It is evident that across experimental groups and the SECG, all participants exhibited positive adaptations in all the outcomes analysed, except for a single man in the SCG who experienced a 1 cm increase in WC. However, in the case of the ICG, notable variations were observed. Specifically, 40% of both men and women saw an increase in WC, while 60% of both genders exhibited an increase in BPs. Furthermore, 90% of women in the ICG experienced an increase in BPd.

Table 3 shows the energy intake recorded in the 3-day diaries administered to the participants before and after the intervention. On average (across the three days), men exhibited a reduction in energy intake from baseline to post-intervention, with decreases of 699.1 kcal in the SCG (pre: 2923.2; post: 2224.2 kcal), 682.5 kcal in the BCG (pre: 2932.5; post: 2250.0 kcal), 625.9 kcal in the SECG (pre: 2958.6; post: 2305.7 kcal), and 73 kcal in the ICG (pre: 2933.8; post: 2860.9 kcal). For women, the respective reductions were 421.8 kcal in the SCG (pre: 2436.9; post: 2015.1 kcal), 426.1 kcal in the BCG (pre: 2460.5; post: 2034.4 kcal), 397.6 kcal in the SECG (pre: 2420.6; post: 2023.1 kcal), and 95.7 kcal in the ICG (pre: 2437.3; post: 2341.6 kcal).

**TABLE 3:** Descriptive statistics (mean ± standard deviation) for the three-day energy intake (kcal) diary at baseline and post-intervention.

	Pre-intervention			Post-intervention			Post-pre (%)			Overall results (post-pre)
	Day 1 (kcal)	Day 2 (kcal)	Day 3 (kcal)	Day 1 (kcal)	Day 2 (kcal)	Day 3 (kcal)	Day 1	Day 2	Day 3	
**SCG**										

Men	2900.8 ± 111.1	2977.8 ± 127.5	2891.1 ± 124.7	2231.2 ± 99.3	2231.4 ± 64.4	2209.9 ± 56.4	-23.1	-25.1	-23.6	Men vs. women *p* < 0.001
	
Women	2437.7 ± 93.4	2431.7 ± 65.5	2441.3 ± 71.4	2010.6 ± 65.4	2015.2 ± 80.5	2019.6 ± 64.7	-17.5	-17.1	-17.3	

**BCG**										

Men	2942.8 ± 125.1	2931.5 ± 73.3	2923.2 ± 88.0	2290.8 ± 144.3	2223.9 ± 104.1	2235.3 ± 95.2	-22.2	-24.1	-23.5	Men vs. women *p* < 0.001
	
Women	2472.9 ± 116.4	2470.7 ± 79.4	2437.9 ± 88.8	2009.7 ± 130.8	2059.4 ± 127.8	2034.1 ± 115.7	-18.7	-16.6	-16.6	

**SECG**										

Men	2958.1 ± 130.3	3011.9 ± 68.2	2905.9 ± 72.0	2255.0 ± 95.2	2380.7 ± 299.6	2281.4 ± 125.1	-23.8	-21.0	-21.5	Men vs. women *p* < 0.001
	
Women	2452.0 ± 47.4	2426.9 ± 63.3	2382.9 ± 92.5	1932.4 ± 85.3	2065.9 ± 83.8	2070.9 ± 158.4	-21.2	-14.9	-13.1	

**ICG**										

Men	2969.9 ± 130.4	2926.6 ± 86.9	2905.1 ± 99.9	2817.9 ± 150.8	2914.1 ± 98.4	2850.7 ± 103.9	-5.1	-0.4	-1.9	Men vs. women *p* = 0.590
	
Women	2391.7 ± 85.7	2439.1 ± 96.2	2481.2 ± 64.3	2305.9 ± 142.8	2415.0 ± 147.6	2304.1 ± 83.0	-3.6	-1.0	-7.1	

SCG: soccer conditioned games; BCG: basketball conditioned games; SECG: self-exercise control group; ICG: inactive control group

Significant interactions were found between group and sex regarding the post-pre mean values of energy intake (F = 75.917; *p* < 0.001; = 0.866). Men experienced significantly greater reductions in average energy intake (post-pre) compared to women in the SCG (men: –699 kcal vs. women: –421 kcal; *p* < 0.001), BCG (men: –682 kcal vs. women: –426 kcal; *p* < 0.001), and SECG (men: –666 kcal vs. women: –398 kcal; *p* < 0.001). However, no significant differences were observed in the ICG (men: –73 kcal vs. women: –96 kcal; *p* = 0.590).

**FIG. 4:**
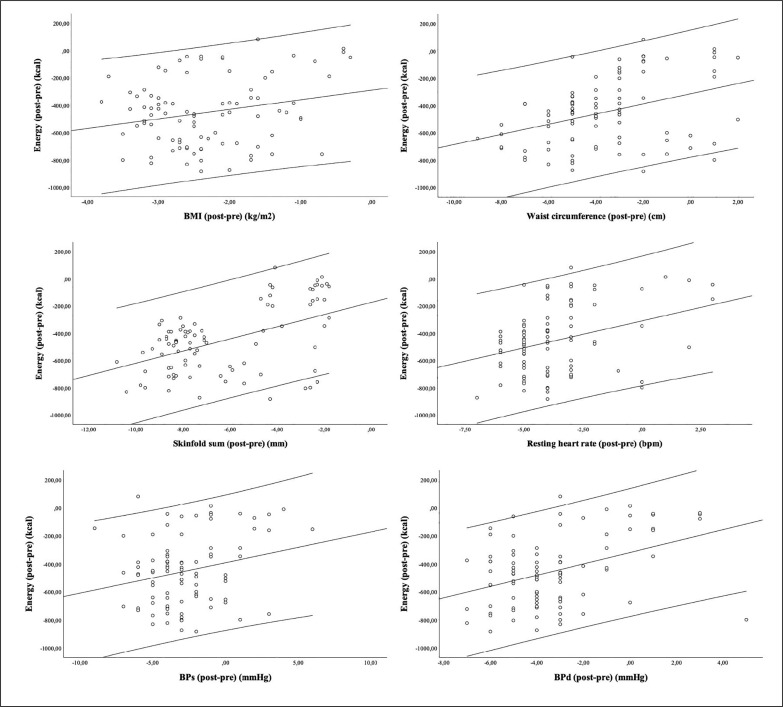
Relationships between post-pre energy intake and adaptations (post-pre) on anthropometric, body composition and physiological variables. Legend: RHR: resting heart rate; BPs: systolic blood pressure; BPd: diastolic blood pressure; SS: skinfold sum; WC: waist circumference; BMI: body mass index; BF: body fat.

The Pearson-product correlation test revealed significant correlations between post-pre mean values of energy intake and the adaptations (post-pre) in BMI (r = 0.231 [95%CI: 0.023;0.417]; *p* = 0.029), WC (r = 0.373 [95%CI: 0.178;0.537]; *p* < 0.001), skinfold sum (r = 0.483 [95%CI: 304;0.626]; *p* < 0.001), RHR (r = 0.329 [95%CI: 0.129;0.500]; *p* = 0.002), BPs (r = 0.239 [95%CI: 0.032;0.424]; *p* = 0.023) and BPd (r = 0.405 [95%CI: 0.214;0.563]; *p* < 0.001). No significant relationships were found between post-pre values of energy intake and adaptations in body fat (r = 0.194 [95%CI: –0.015;0.384]; *p* < 0.001). The relationships can be observed in the scatterplot shown in Figure 4.

## DISCUSSION

The study aimed to examine the effects of recreational soccer and basketball conditioned games, self-exercise, and inactivity on health measures in sedentary overweight and obese individuals. Significant improvements in body composition and cardiovascular health were observed across the SCG, BCG, and SECG groups when compared to the ICG, regardless of sex. Furthermore, nearly all participants in the experimental groups and SECG exhibited significant improvements in their outcomes, while those in the ICG experienced heterogeneous variations in their outcomes.

The inter-group analysis, considering the variations (post-pre) observed within each group, indicated a beneficial effect of both the SCG and the BCG in terms of the extent of beneficial effects on BMI, WC, skinfold sum, RHR, and BPs. No significant differences were observed between the SCG and BCG. This evidence suggests the effectiveness of recreational team sports in improving body composition and physiological/cardiovascular parameters in overweight and obese populations, as previous studies have also shown [16, 17]. However, while the SECG also experienced significant improvements, their extent was not similar to the experimental groups. Possibly, the intensity parameters experienced or the volume of practice could be of high interest to match the demands imposed by soccer and basketball activities, as they often require a high level of commitment to exercise due to the intense efforts commonly reported in smallformat games like those applied in this study [32].

Regarding the within-group pre-post changes, the SCG and BCG groups showed significant improvements in BMI, body fat percentage, and WC. This finding aligns with the existing body of evidence that shows the effectiveness of recreational team sports in improving anthropometric and body composition measures in sedentary individuals [33, 34]. Such improvements could be attributed to the high-intensity and intermittent nature of soccer and basketball, which have been shown to yield significant cardiovascular and metabolic acute and chronic adaptations over time [35]. The fact that these improvements were more pronounced in the SCG and BCG compared to the SECG suggests that a more structured and socially motivating environment such as recreational team sports can play an important role in physical activity. Previous studies have shown that, when compared to other analytical training regimens, such as in the SECG, recreational team sports are associated with longer-term practice in sedentary individuals [36, 37].

Although self-exercise has been found to be as effective as SCG and BCG in improving the skinfold sum, RHR, BPs, and BPd measures, its long-term effects on such variables remain questionable [38]. On the other hand, the practice of recreational team sports, such as soccer and basketball, has been found to produce both acute and chronic health adaptations [13]. For instance, a previous systematic review showed that recreational soccer practice results in significant cardiovascular and muscular adaptations and significant health improvements over time in sedentary individuals [14]. Furthermore, as found in previous studies, maintaining regular practice of any form of physical activity, such as in recreational team sports, or autonomous exercise, is inevitably more effective to ensure improvements in overall health than being inactive [39, 40].

The improvements in health measures observed in the present study did not differ significantly between male and female participants, indicating the potential of both recreational soccer and basketball practice as effective physical activity alternatives to analytical approaches, such as self-selected exercises. Although significant differences were previously found between sexes for technical soccer measures, such as passing and shooting accuracy [41, 42], there is little to no evidence of differences in body composition and cardiorespiratory health measures between sexes after a recreational soccer and/or basketball intervention [43]. Notwithstanding the lack of evidence on sex differences, the present study showed that both sexes can effectively improve their overall health, such as by decreasing their body fat, BMI, and WC, and improving their RHR, BPs, and BPd.

Despite our study findings, there were some limitations. The main limitation was that the participants were exposed to only 12 weeks of intervention. This could have limited the observation of greater improvements in body composition and vascular health. A longer intervention period might have provided deeper insights into the longterm effects of recreational soccer and basketball conditioned games. Furthermore, the absence of load monitoring throughout the interventions produces a gap between the total work done and the extent of the health improvements observed after the intervention. Future studies must consider controlling for training loads to analyse the threshold needed to observe positive adaptations to both recreational soccer and basketball conditioned games.

As for practical implications, this study indicates that engaging in recreational team sports three times a week, with a focus on smallsided games, effectively enhances the body composition and cardiovascular health of overweight and obese individuals. Consequently, policymakers should consider implementing community-based team sports training programmes to capitalize on these positive effects among such populations. Moreover, emphasizing the enjoyment derived from engaging in team sports may foster sustained involvement, surpassing that of individual sports activities, especially for those individuals who may not be motivated towards such sports.

## CONCLUSIONS

The current experimental study showed that overweight and obese inactive young adults can significantly benefit in terms of anthropometrics, body composition, and cardiovascular health by engaging in recreational soccer and basketball through small-sided games. These improvements were significantly greater compared to participants who maintained their inactive lifestyle or those in control groups who initiated self-exercise. These differences appeared to be independent of sex, as no interaction was found between group and sex.

However, it was observed that following the experimental intervention, participants exhibited increased dietary restraint. By regulating their energy intake, it was possible to observe a positive and significant association between the magnitude of reductions in energy intake and improvements in anthropometrics, body composition, and cardiovascular health. These relationships suggest that interactions among lifestyle factors such as physical exercise and diet may play a role in explaining the observed positive outcomes in overweight and obese men and women.
